# [Corrigendum] Hydrogen gas post‑conditioning alleviates cognitive dysfunction and anxiety‑like behavior in a rat model of subarachnoid hemorrhage

**DOI:** 10.3892/etm.2024.12541

**Published:** 2024-04-17

**Authors:** Jing-Hua Song, Hong-Yan Jia, Tian-Peng Shao, Zhi-Bao Liu, Yuan-Ping Zhao

Exp Ther Med 22:1121, 2021; DOI: 10.3892/etm.2021.10555

Following the publication of the above article, an interested reader drew to the authors’ attention that, in Fig. 5B on p. 7, concerning the DAPI-stained cells, the ‘DAPI / SAH’ and ‘DAPI / SAH+H2’ data panels appeared to be overlapping, such that the data may have been derived from the same original source where they were intended to have shown the results from differently performed experiments. The authors have re-examined their original data, and realized that the data in this figure had inadvertently been assembled incorrectly.

The revised version of Fig. 5, now incorporating the correct data for the SAH+H2’ data panel in Fig. 5B, is shown on the next page. Note that the error made in assembling this figure did not have a significant impact on either the results or the conclusions reported in the paper. All the authors agree with the publication of this corrigendum, and are grateful to the Editor of *Experimental and Therapeutic Medicine* for allowing them the opportunity to publish this. Moreover, they apologize to the readership for any inconvenience caused.

## Figures and Tables

**Figure 5:**
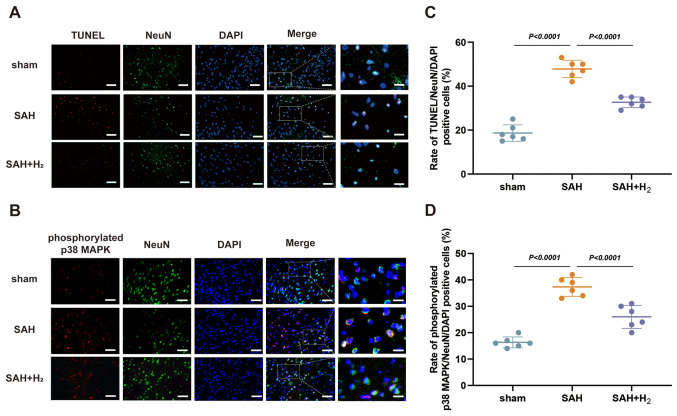
H2 post-conditioning reduces neuronal apoptosis and alters SAH-induced phosphorylated-p38 MAPK in neurons. (A) Representative photomicrographs of NeuN and TUNEL staining (NeuN, green; TUNEL, red; DAPI, blue), showing apoptotic neurons in the vmPFC on 24 h after SAH (n=6). Scale bar, 50 or 15 µm. (B) Representative photomicrographs of NeuN and phosphorylated p38 MAPK staining (NeuN, green; p38 MAPK, red; DAPI, blue), showing phosphorylated p38 MAPK-positive neurons in the vmPFC on 24 h after SAH (n=6). Scale bar, 50 or 15 µm. (C) Rate of apoptotic neurons in the vmPFC, induced by the indicated stimuli. (D) Rate of phosphorylated p38 MAPK-positive neurons in the vmPFC, induced by the indicated stimuli. SAH, subarachnoid hemorrhage; vmPFC, ventromedial prefrontal cortex; NeuN, neuronal nuclei.

